# Emerging Role of MiR-192-5p in Human Diseases

**DOI:** 10.3389/fphar.2021.614068

**Published:** 2021-02-23

**Authors:** Fu-jia Ren, Yao Yao, Xiao-yu Cai, Guo-ying Fang

**Affiliations:** ^1^ Department of Pharmacy, Hangzhou Women’s Hospital (Hangzhou Maternity and Child Health Care Hospital), Hangzhou, China; ^2^ Department of Pharmacy, Women’s Hospital School of Medicine, Zhejiang University, Hangzhou, China; ^3^ Department of Pharmacy, Hangzhou First People’s Hospital, Hangzhou, China

**Keywords:** miR-192-5p, human diseases, Cancers, respiratory system, digestive system

## Abstract

MicroRNAs (miRNAs) are a type of small non-coding RNAs that play an essential role in numerous biological processes by regulating the post-transcriptional expression of target genes. Recent studies have demonstrated that miR-192-5p, a member of the miR-192 family, partakes in several human diseases, especially various cancers, including cancers of the lung, liver, and breast. Importantly, the levels of miR-192-5p are abundant in biofluids, including the serum and urine, and the exosomal levels of miR-192-5p in circulation can aid in the diagnosis and prognosis of various diseases, such as chronic hepatitis B (CHB) infection disease. Notably, recent studies suggest that miR-192-5p is regulated by long noncoding RNAs (lncRNAs) and circular RNAs (circRNAs). However, there are no comprehensive overviews on the role of miR-192-5p in human diseases. This review discusses the significant studies on the role of miR-192-5p in various human diseases, with special emphasis on the diseases of the respiratory and digestive systems.

## Introduction

MicroRNAs (miRNAs) are short (18–25 nt), noncoding, single-stranded RNAs, that regulate the expression of target genes by binding to the 3′-untranslated region (UTR) or open reading frames (ORFs) of the target mRNA for inducing translational repression or mRNA degradation in animals (Jonas and Izaurralde, 2015). However, some studies have reported that certain miRNAs facilitate gene expression (Place et al., 2008). In particular, many studies have demonstrated that miRNAs play a role in numerous cellular processes, including cellular differentiation, oxidative stress, apoptosis, and autophagy. Increasing evidence suggests that a large number of miRNAs are dysregulated in various human diseases, especially cancers. These studies indicate that miRNAs could either act as oncogenes or tumor suppressors, despite the fact the miRNAs are generally downregulated in tumors, compared to normal tissues (Lu et al., 2005). Meaningfully, the miRNAs present in biological biofluids are potential non-invasive diagnostic and prognostic biomarkers of various human diseases. Moreover, accumulating studies highlight that exosomal miRNAs in biofluids as essential participants in tumorigenesis (Ingenito et al., 2019). Due to the nature delivery systems of exosomes between cells, some therapeutic miRNAs can be appropriately packaged in exosomes to target specific recipient cells. Intriguingly, increasing reports indicate that the expression levels of miRNAs are regulated by long non-coding RNAs (lncRNAs) and circular RNAs (circRNAs) (Cai et al., 2019; Liu et al., 2019; Sun et al., 2020). Noteworthily, cytoplasmic lncRNAs or circRNAs frequently possess potential sites for binding to certain miRNAs, and can repress miRNA function by acting as miRNA sponges in tumor development (Shi et al., 2013; Kulcheski et al., 2016). Therefore, miRNAs, as post-transcriptional gene regulators, have therapeutic and application potential.

**FIGURE 1:**
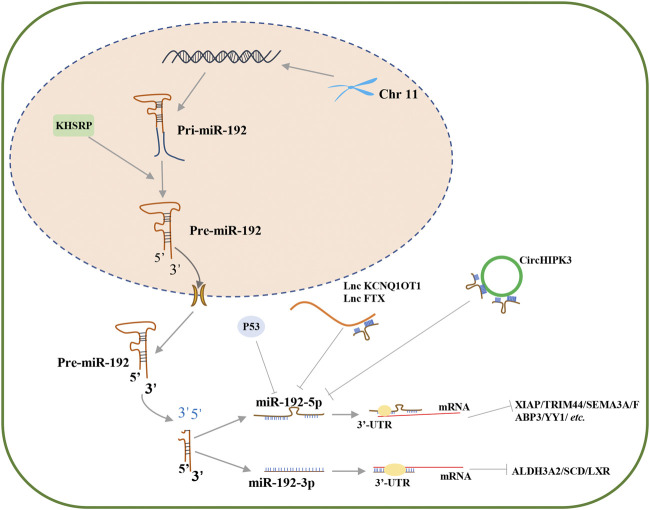
The biogenesis and regulation of miR-192-5p. Pre-miR-192 could produce two mature miRNA transcripts, miR-192-5p and miR-192-3p, each of which can regulate gene expression by targeting 3′-UTR of mRNA, such as XIAP, TRIM44, SEMA3A, FABP3, and YY1 for miR-192-5p, and ALDH3A2, SCD and LXR for miR-192-3p. In cell nucleus, KHSRP can promote maturation of pri-miR-192, thereby facilitating the production of miR-192-5p. In cytoplasm, P53 and noncoding RNAs, such as circHIPK3, lnc KCNQ1OT1 and lnc FTX, can inhibit the expression of miR-192-5p.

It is worth mentioning that miR-192-5p is a conserved miRNA, which is abundant in the liver, and plays important roles in numerous hepatic disorders, including chronic hepatitis B (CHB), drug-induced liver injury, nonalcoholic fatty liver disease (NAFLD), and hepatocellular carcinoma (HCC) (Nielsen et al., 2018; Roy et al., 2016; Wen et al., 2015). Importantly, increasing evidence suggests that miR-192-5p is closely involved in different physiological and pathological processes, especially in the incidence of cancer. Moreover, exosomal miR-192-5p in biological fluids may be a potential biomarker of disease progression, such as NAFLD (Liu et al., 2019). Interestingly, the expression of miR-192-5p is regulated by diverse factors, including p53 and TGF-β (Chen et al., 2016; Puppo et al., 2016). For instance, Puppo and coworkers reported that TGF-β represses the expression of KHSRP, a component of the ribonucleoprotein complex, which controls the maturation of miR-192-5p and promotes the expression of some epithelial-to-mesenchymal transition (EMT)factors (Figure 1) (Puppo et al., 2016). Besides, lncRNAs and circRNAs can also act as upstream regulators that modulate the expression of miR-192-5p (Cai et al., 2019; Sun et al., 2020). A recent study by Sun and coworkers revealed that the opposite strand/antisense transcript 1 (KCNQ1OT1) lncRNA inhibits sepsis-induced myocardial injury by regulating the miR192-5p/X-linked inhibitor of apoptosis (XIAP) axis (Sun et al., 2020). Zhao and coworkers demonstrated that the FTX lncRNA promotes the progression of colorectal cancer (CRC) by targeting the miR-192-5p/EIF5A2 axis (Zhao et al., 2020b). Cai and coworkers reported that circHIPK3 promotes hyperglycemia and insulin resistance by acting as a sponge for miR-192-5p, which subsequently induces the expression of the forkhead box O1 (FOXO1) protein (Cai et al., 2019). Therefore, miR-192-5p is a critical miRNA for the progression of human diseases, which can be regulated by other vital regulatory molecules and subsequently affect the expression of downstream genes (Figure 1).

**FIGURE 2:**
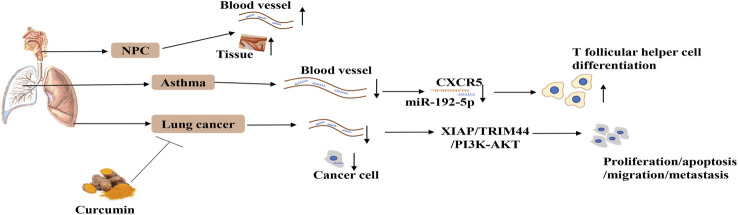
The expression and regulatory mechanism of miR-192-5p in respiratory system. MiR-192-5p is reduced in serum of human asthma, NPC and lung cancer, as well as NPC tissues and lung cancer cell, which can influence diseases progression by targeting CXCR5, XIAP, TRIM44, and PI3K-AKT. Curcumin can promote the expression of miR-192-5p.

**FIGURE 3:**
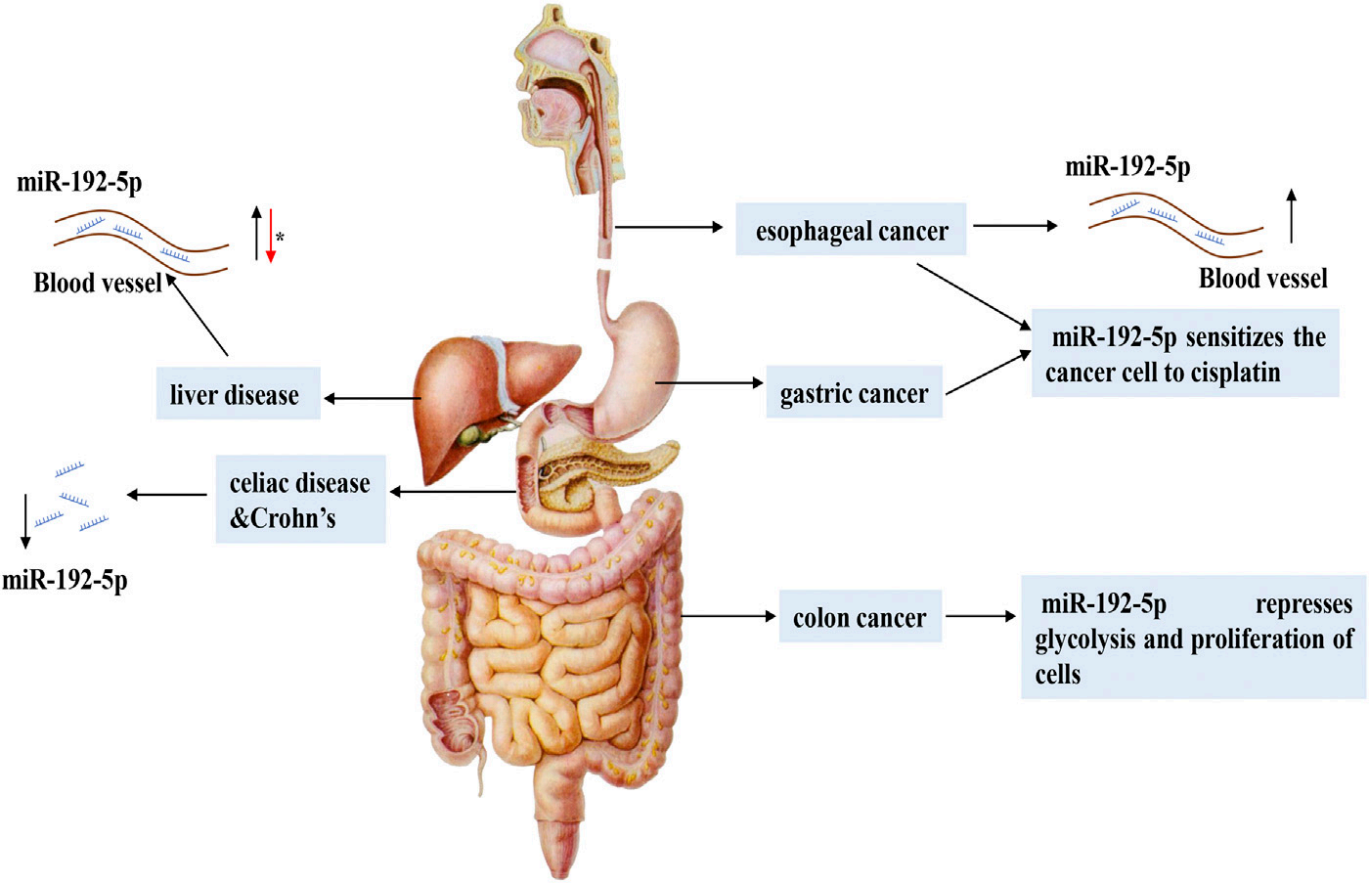
The expression and regulatory mechanism of miR-192-5p in digestive system. In different digestive system diseases, the expression of miR-192-5p is flexible. In various liver diseases, miR-192-5p exhibits different expression levels and exerts versatile function. The asterisk (*) represents the controversial role of miR-192-5p.

This review systematically summarizes recent studies on the functions and mechanisms underlying the role of miR-192-5p in different physiological processes and human diseases (Table 1). The potential regulatory role of miR-192-5p in diseases of the respiratory (Figure 2) and digestive systems (Figure 3) are emphasized, which would enable a comprehensive understanding of the role of miR-192-5p in the occurrence of certain diseases and present novel directions for future research.

**TABLE 1:** MiR-192-5p in different human diseases. The asterisk (*) represents controversial.

Human system	Disease type	Role	Target	References
Respiratory	Asthma	Represses the differentiation of T follicular helper cell	CXCR5	Zhang et al. (2018), Lou et al. (2020), and Yamamoto et al. (2012)
		Alleviates airway remodeling and autophagy	MMP-16 and ATG7	
	Lung cancer	Suppresses the tumor growth in NSCLC cell lines	XIAP	Ye et al. (2015), Zou P et al. (2019), Jin et al. (2015), and Kumar et al. (2020)
		Inhibits the proliferation, migration and invasion of lung cancer cells	TRIM44	
Digestive	HCC*	Promotes proliferation and metastasis of HCCLM3 cells	SEMA3A	Yan-Chun et al. (2017), Gu et al. (2019), and Zhu et al. (2019)
		Inhibition of miR-192-5p promotes CSC populations	PABPC4	
		Inhibits the progression of HCC.	TRIP13	
	NAFLD	Inhibit lipid synthesis	SCD-1	Liu XL et al. (2017), Liu et al. (2019), Lee et al. (2019), and Pirola et al. (2015)
		Promote the M1 macrophage polarization	Rictor	
	Liver injury	Positively correlated with injury degree	Zeb2	Roy et al. (2016), Zhang et al. (2020), Yan et al. (2014), and Church et al. (2016)
		Promote DMF-induced hepatotoxicity	NOB1	
	Colon cancer	Represses cell glycolysis	SRPX2	Zhao et al. (2018), Huang et al. (2020)
		Inhibits cell proliferation	RhoA-ROCK-LIMK2 pathway	
	Gastric cancer	Reverses cisplatin resistance	ERCC3 and ERCC4	Xie et al. (2019), Tavakolian et al. (2020)
Circulation	Arrhythmic diseases	Aggravates the arrhythmia	SCN5A	Zhao et al. (2015)
	Myocardial injury	Promotes apoptosis of cardiomyocyte	FABP3	Zhang et al. (2017), Sun et al. (2020)
Urinary	Kidney injury	Promotes collagen deposition in diabetic kidney	SIP1	Kato et al. (2007), Baker et al. (2019), and Chen et al. (2016)
		Protects kidney against hypertension	ATP1B1	
		Inhibition of miR-192-5p alleviates VAN-induced AKI.		
Reproductive	Breast cancer	Negatively correlates with ERα expression	LY6K	Kim et al. (2016), Chen et al. (2018), Fang et al. (2018), and Zhang et al. (2019)
	Prostate cancer	Promotes proliferation of prostate cancer cells	UBASH3B	Chen ZJ et al. (2019), Wang et al. (2019)
Endocrine	Diabetes	Represses insulin resistance	FOXO1	Cai et al. (2019), Argyropoulos et al. (2015), Jaeger et al. (2018), Mo et al. (2017), Nunez Lopez et al. (2019), and Wu et al. (2015)
Nervous	Depression	Rescues cognitive impairment and repair neural function	Fbln-2	Tang et al. (2019)
	Left sciatic nerve injury	Promotes neuronal apoptosis	XIAP	Liu et al. (2020)
	PD	Represses cell viability and induce apoptosis	Unknown	Kang et al. (2019)
Motor	RA	Delays inflammatory response	RAC2	Zheng et al. (2020), Li et al. (2017)
		Inhibits the proliferation and induce apoptosis of human rheumatoid arthritis FLS	caveolin 1	
	Osteosarcoma	Represses the proliferation, migration and invasion of osteosarcoma cells	USP1	Zhou S et al. (2018)

### Biogenesis of miR-192-5p

Data from the miRbase database reveal that there are approximately 80 different human miRNA precursors, that produce two mature miRNAs, namely, the 5′ and 3′ strands, with different seed sequences and target mRNAs to which they bind. Human miR-192 is derived from a coding gene located on chromosome 11, which produces two mature transcripts, miR-192 (miR-192-5p) and miR-192* (miR-192-3p) (Bartel, 2004; Krattinger et al., 2016). Both miR-192-5p and miR-192-3p inhibits the translation of target mRNAs by targeting the 3′-UTR in different biological processes (Figure 1).

Numerous studies have investigated the roles of miR-192-5p; however, the functions of miR-192-3p are poorly explored. The study by Mysore and coworkers emphasized the important role of miR-192-3p in adipocyte differentiation and lipid homeostasis. First, they found an interesting phenomenon that miR-192-3p in the visceral adipose tissue (VAT) of obese patients is negatively correlated with the levels of serum triglyceride (TG). Further mechanism studies have demonstrated miR-192-3p represses the adipocytic differentiation of human Simpson–Golabi–Behmel syndrome (SGBS) adipocytes by targeting aldehyde dehydrogenase 3 family member A2 (ALDH3A2) and stearoyl coenzyme A desaturase 1 (SCD), which is indicated by a significant reduction in the TG content and the decreased expression of the adipocyte marker proteins, aP2 and perilipin 1 (Mysore et al., 2016). Additionally, miR-192-5p and miR-192-3p have a synergistic role in downregulating the expression of farnesoid X receptor (FXR) and the target genes of FXR in the liver and colon cancer-derived cell lines. However, miR-192 has differential effects on the expression of FXR in Huh7 and Caco-2 cells (Krattinger et al., 2016). Sometimes they work synergistically together and sometimes individually, underscoring their different relationships to each other in different pathological conditions. Although this review primarily discusses the role of miR-192-5p in human diseases, the additive effect of the 3′-transcript should not be overlooked.

### Physiological Roles of miR-192-5p

It has been reported that miR-192-5p is abundant in the liver, where it promotes the development of the liver, cellular *trans*-differentiation, and coordinates energy metabolism (Raut and Khanna, 2016). Moreover, the levels of miR-192-5p are negatively correlated with the expression of the multidrug transporter, ATP binding cassette transporter subfamily C member 3 (ABCC3), in the human intestine, signifying that miR-192-5p may influence drug absorption (Bruckmueller et al., 2017). We can suspect that miR-192-5p may play an important role in the normal liver, the largest digestive glands, and intestines in the human body, but the current studies are not sufficient and more data are needed to support and improve this inference.

A recent miRNA profiling study suggested that miR-192-5p may be implicated in the developmental competence of female oocytes, indicating that miR-192-5p has a potential role in the reproductive system (Woo et al., 2018). It is widely acknowledged that uterine receptivity is vital for embryo implantation, and it starts with the transformation of the luminal epithelium (Murphy, 2004). Interestingly, one study by Liang and coworkers reported that miR-192-5p is downregulated rapidly in the uteri of mice before implantation, and retains at low expression levels during and after implantation. By performing *in situ* hybridization assay, they found miR-192-5p is expressed in the endometrial epithelium, and dysregulation of miR-192-5p disturbed the performance of the luminal epithelium, leading to inadequate receptivity. This study emphasized the physiological role of miR-192-5p in the mouse uterus is to keep the nonreceptive state of epithelial cells and impede their transformation to the receptive state (Liang et al., 2020). Therefore, miR-192-5p may be a vital regulator in the orderly process of the reproductive system.

Researchers have also demonstrated that miR-192-5p is involved in regulating blood pressure and heart rate. Thus, miR-192-5p plays a crucial role in the vital biological processes of human physiology. In particular, overwhelming data have reported that miR-192-5p regulates oxidative stress, cellular proliferation, apoptosis, and inflammatory responses (Caserta et al., 2016; Fuschi et al., 2017). Given its important role in human physiological processes and cellular processes, the dysregulation of miR-192-5p may contribute to the genesis of human diseases.

## MiR-192-5p in Human Diseases

### Diseases of the Respiratory System

#### Asthma

Asthma is a common respiratory disorder, characterized by variable respiratory symptoms and variable airflow limitation, that affects around 334 million people worldwide (Papi et al., 2018). Miserably, the prognosis of asthma remains poor, despite the application of several strategies to the treatment of asthma (Gibeon and Menzies-Gow, 2013). Fortunately, increasing evidence suggests that miR-192-5p could be a potential target for the treatment of asthma. In 2012, Yamamoto and coworkers first identified that the levels of miR-192 are significantly downregulated in the peripheral blood of asthmatic individuals undergoing an allergen inhalation challenge compared to those of healthy controls (HCs) (Yamamoto et al., 2012), suggesting a potential role of miR-192-5p in asthma. Subsequently, another study demonstrated that the levels of miR-192 are lower in children with asthma compared to those of HCs, and further experimental studies confirmed that miR-192 represses the differentiation of follicular T-helper cells by targeting C-X-C chemokine receptor type 5 (CXCR5) in childhood asthma (Zhang et al., 2018). Moreover, Lou and coworkers demonstrated that the levels of miR-192-5p are decreased in asthmatic mice, and the overexpression of miR-192-5p alleviates airway remodeling and autophagy in asthma by targeting matrix metalloproteinase-16 (MMP-16) and autophagy-related 7 (ATG7) *in vitro* and *in vivo* (Lou et al., 2020). In general, these four studies revealed miR-192-5p may be a potential regulator in the progression of asthma, and further studies are needed to elucidate the deep mechanisms.

#### Nasopharyngeal Carcinoma

Nasopharyngeal carcinoma (NPC) is an epithelial carcinoma originating from the nasopharyngeal mucosal lining, which is regarded as a considerable health burden in low resource countries, exhibiting unbalanced geographical distribution, including Southeastern Asia (Chua et al., 2016). There are approximately 129000 new cases of NPC in 2018, according to the International Agency for Research on Cancer (Chen YP et al., 2019). Epstein–Barr virus (EBV) infections are correlated with the pathogenesis of NPC, and detection of plasma EBV DNA has been utilized for population screening, prognostication, predicting treatment response for therapeutic adaptation, and disease surveillance (Chen YP et al., 2019). In 2020, a study on miRNA profiling in the serum of patients with NPC revealed that 30 miRNAs were aberrantly expressed in the serum. Among these, miR-192-5p was found to be upregulated in the serum and tissues of patients with NPC, however, there was no significant difference in miR-192-5p expression in the exosomes. To study the diagnostic applications of identified miRNAs, researchers found that the expression of miR-192-5p is downregulated in EBV-positive patients, but upregulated in EBV-negative patients, compared to that of normal controls (NCs), suggesting that the status of EBV infection can influence miR-192-5p expression. Using a logistic regression model for evaluating the prediction probability of NPC, researchers have constructed a panel of five miRNAs (let-7b-5p, miR-140-3p, miR-192-5p, miR-223-3p, and miR-24-3p), which has significantly high sensitivity and specificity for discriminating patients with NPC from healthy individuals. However, survival analysis revealed that the level of miR-192-5p is less related to the clinical outcomes of the patients. Therefore, further studies are necessary for elucidating the function and mechanism underlying the role of miR-192-5p in NPC for exploring a valuable biomarker for predicting NPC (Zou et al., 2020).

#### Lung Cancer

Recent epidemiology data demonstrated that lung cancer is still the leading cause of cancer morbidity and mortality worldwide, with 2.1 million new lung cancer cases and 1.8 million deaths predicted in 2018, of which non-small cell lung cancer (NSCLC) accounts for approximately 80% of lung cancer (Jemal et al., 2010; Bray et al., 2018). While investigating the anticancer effect of curcumin, a traditional Chinese medicine derived from the medical plant *Curcuma longa*, in 2015, Ye and coworkers unexpectedly found that miR-192-5p functions as a tumor suppressor in NSCLC. The authors initially confirmed that curcumin could inhibit the growth and induce apoptosis in two NSCLC cell lines, H460 and A427, at all the tested doses. They further demonstrated the *in vivo* anti-cancer effects of curcumin using a nude mouse tumorigenicity assay. Using a miRNA array and qPCR, the authors identified that miR-192-5p was the most responsive miRNA, and its expression was increased by more than a four-fold following treatment with curcumin. The study further revealed that miR-192-5p suppresses tumor growth in NSCLC cell lines by inhibiting the expression of XIAP (Ye et al., 2015). Interestingly, the authors observed that curcumin promotes the expression of miR-192-5p via p53-dependent mechanisms (Ye et al., 2015). Subsequently, another research team confirmed that curcumin represses cellular proliferation and promotes the apoptosis of human NSCLC cells by upregulating the expression of miR-192-5p, which subsequently suppresses the activity of PI3K/Akt (Jin et al., 2015). The study further demonstrated that curcumin alters the expression of miR-192-5p, which subsequently suppresses the expression of c-Myc and the Wnt/β-catenin pathway in two NSCLC cell lines, A427 and A549 (Pan et al., 2020). It is widely acknowledged that approximately 50%–70% of patients with lung cancer suffer from bone metastasis. In 2019, Zou and coworkers demonstrated that the expression of miR-192-5p is downregulated in the serum of patients with lung cancer and lung cancer cell lines, while the expression of tripartite motif 44 (TRIM44) shows an opposite tendency. Further studies have demonstrated that miR-192-5p inhibits the proliferation, migration, and invasion of lung cancer cells by targeting TRIM44 *in vitro* and *in vivo*, particularly by repressing bone metastasis (Zou P et al., 2019). Coincidently, small RNA sequencing by Kumar and coworkers demonstrated that the expression of miR-192-5p is reduced in the serum of Indian patients with NSCLC, and further study revealed that the expression of miR-192-5p is correlated with the sex of the patient (Kumar et al., 2020). Conclusively, miR-192-5p may be a potential therapeutic biomarker of NSCLC and a potential target of turmeric, with good development potential.

It is worth mentioning that NSCLC is a heterogeneous disease, which can be divided into 2 broad histologic subtypes, namely, the squamous cell carcinoma (LUSC) and adenocarcinoma (LUAD) subtypes (Lim et al., 2020). Recently, a plasma exosome miRNA profiling study performed on six healthy individuals, six patients with LUAD before surgery, and six patients after 7 days of surgery indicated that the expression of miR-192-5p is sharply increased in the plasma exosomes of patients with LUAD, where a high abundance of miR-192-5p is indicative of poor survival rates. Besides, the study demonstrated that miR-192-5p is significantly downregulated post-surgery, suggesting a tumor promotive role of miR-192-5p in LUAD (Xue et al., 2020). These results are controversial owing to the paradox between the inference of this study with that of previous studies on the anti-cancer effect of miR-192-5p in NSCLC. Therefore, further studies are urgently required for elucidating the potential role of miR-192-5p in lung cancer.

### Diseases of the Digestive System

#### Hepatic Disorders

**FIGURE 4:**
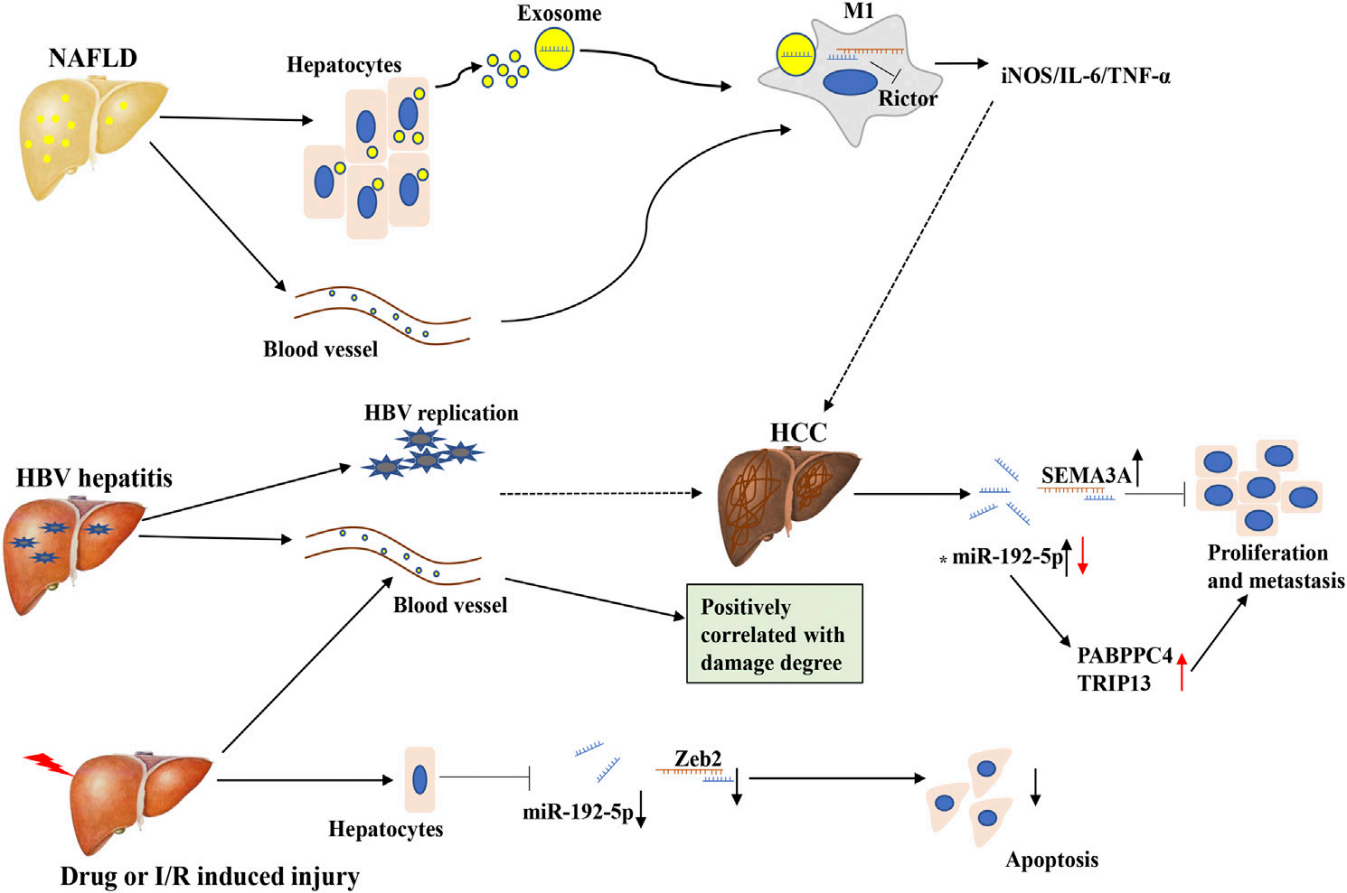
The expression and regulatory mechanism of miR-192-5p in different liver diseases. In NAFLD, miR-192-5p is increased in serum exosome, and exosomal miR-192-5p derived from hepatocytes promote the polarization of inflammatory macrophage. In HBV hepatitis, drug or I/R induced liver jury, circulating miR-192-5p is increased, which can target different molecules. In HCC, the expression of miR-192-5p is controversial. Full line represents what actually happens, dotted line represents what might happen. The asterisk (*) represents the controversial role of miR-192-5p.

It has been reported that miR-192-5p is abundantly expressed in hepatic tissues, and is regarded as a potential biomarker for various hepatic disorders (Figure 4) (Iguchi et al., 2017; Bushel et al., 2018; Oda et al., 2018). Previously, an important study demonstrated that the expression of miR-192-5p markedly increases during the development of the human liver, which induces hepatic *trans*-differentiation from human umbilical cord Wharton’s jelly derived mesenchymal stem cells (hUC-MSCs), indicating the crucial role of miR-192-5p in the physiological processes of the human liver (Tzur et al., 2009; Raut and Khanna, 2016). Chronic hepatitis B (CHB) infection leads to the development of severe liver diseases, such as hepatic cirrhosis and HCC. In 2014, researchers first identified that miR-192-5p is overexpressed in both the sera and HBsAg-particles of CHB by miRNA profiling analysis of inactive-carriers (IC) and patients of CHB, suggesting an emerging role of miR-192-5p in CHB (Brunetto et al., 2014). Subsequently, another research team identified that miR-192-5p is present in hepatoma-derived extracellular vesicles and is abundantly expressed in HBeAg-positive patients compared to HBeAg-negative patients, signifying miR-192-5p is associated with HBeAg-status (van der Ree et al., 2017). Moreover, in 2018, one study by Nielsen and coworkers demonstrated that the replication of HBV is correlated with the *in vitro* expression of miR-192-5p in the HepG2 cell model system, and overexpression of miR-192-5p by mimics reduces the protein level of pro-apoptotic BIM and suppresses endoplasmic reticulum (ER) stress-induced apoptosis in HepG2 cells (Nielsen et al., 2018). Summarily, miR-192-5p may contribute to the development of HBV-related liver diseases by influencing HBeAg-status. However, Tan and coworkers found the serum level of miR-192-5p is significantly reduced in cirrhosis that evolves into hepatocellular carcinoma, during hepatitis B virus infection (Tan et al., 2015). Therefore, we suspected that circulating miR-192-5p is probably a flexible molecule, and contributes to the progression of various HBV-related liver diseases.

According to the GLOBOCAN 2018 database, liver cancer is the sixth most commonly diagnosed cancer and the fourth leading cause of cancer death. HCC (comprising 75%–85% of cases) is the most frequent primary liver cancer, and is closely related to chronic HBV infection (Bray et al., 2018). Notably, the emerging role of miR-192-5p in HCC has been gradually investigated. In 2015, a multicenter three-phase study indicated that miR-192-5p is a potential early serum biomarker for the detection of HCC (Wen et al., 2015). Subsequently, a study by Li and coworkers revealed that miR-192-5p can promote the proliferation and metastasis of the HCC cell line, HCCLM3, by targeting semaphorin 3A (SEMA3A), a potent inhibitor of tumor angiogenesis in different cancers (Yan-Chun et al., 2017). It is widely acknowledged that cancer stem cells (CSCs) are closely related to the initiation, metastasis, recurrence, and chemo-resistance of various tumors, especially in HCC (Oikawa, 2016). In contrast to the research presented above, the expression of miR-192-5p is downregulated in CSC^+^ HCC and the inhibition of miR-192-5p promotes the development of multiple populations of CSCs and CSC-related features by targeting PABPC4 (Gu et al., 2019). Mechanistically, the miR-192 promoter is hyper-methylated, which leads to the transcriptional repression of miR-192-5p in HCC cell lines and primary CSC^+^ HCC (Gu et al., 2019). Besides, Zhu and coworkers reported that miR-192-5p is an upstream regulator of thyroid hormone receptor interactor 13 (TRIP13), a promising oncogene in HCC, by directly targeting the 3′-UTR of TRIP13 mRNA, thereby inhibiting the progression of HCC (Zhu et al., 2019). Interestingly, the expression of miR-192-5p is also regulated by other molecules in HCC, including circRNA, although increasing evidence has demonstrated that numerous molecules are influenced by miR-192-5p (Qiu et al., 2019). As the role of miR-192-5p in HCC is controversial, it is difficult to define miR-192-5p as an oncogene or tumor suppressor. Further studies are necessary for elucidating the function and mechanism underlying the role of miR-192-5p in HCC.

In 2015, a global serum miRNA profiling study revealed that miR-192-5p could be a potential regulator of NAFLD (Pirola et al., 2015). Then, a study by Liu and coworkers demonstrated that miR-192-5p is downregulated in high-fat diet (HFD) induced rat NAFLD, and overexpression of miR-192-5p inhibits lipid synthesis by targeting stearoyl-CoA desaturase 1 (SCD-1) in Huh7 cells exposed to

PA (Liu XL et al., 2017). Interestingly, a recent study the same research team reported that circulating and hepatocyte-derived exosomal miR-192-5p can contribute to the inflammatory response in NAFLD. First, they identified that the levels of miR-192-5p in the serum of NAFLD patients are positively correlated with pathological features of the liver, including AST, ALT, the occurrence of hepatic steatosis, and activity score. Coincidentally, a similar phenomenon was observed in rats with HFD-induced NAFLD. Functionally, the serum levels of miR-192-5p are related to the phenotypic variation of macrophages, and exosomal miR-192-5p secreted by lipotoxic hepatocytes promotes the activation of M1 macrophages (CD11b^+^ CD86^+^) and increases the expression of the M1 marker genes, *iNOS*, *IL-6*, and *TNF-α*. Mechanistically, hepatocyte-derived exosomal miR-192-5p represses the expression of the rapamycin-insensitive companion of mammalian target of rapamycin (Rictor) protein by targeting the 3′-UTR of its mRNA, which subsequently inhibits the activation of AKT and promotes the activation of FOXO1 (Liu et al., 2019). More importantly, a recent study reported that the consumption of blackcurrant (*Ribes nigrum*) in C57BL/6J mice prevents HFD-induced nonalcoholic steatohepatitis (NASH), as indicated by the lower infiltration of macrophages, especially M1 macrophages, and the reduced circulating levels of miR-122-5p and miR-192-5p (Lee et al., 2019). Therefore, miR-192-5p plays an important role in lipid metabolism and inflammation in NAFLD, making it a promising diagnostic target for NAFLD, and further mechanistic studies are needed to perform in order to clarify its meaningful role.

Additionally, miR-192-5p also plays a role in various liver injuries induced by damaging factors. A study by Yan and coworkers on the profile of circulating miRNAs revealed that the levels of miR-192-5p are significantly upregulated in mice after 28 days of exposure to perfluorooctanoic acid (PFOA), a known hepatotoxic compound with industrial and commercial uses (Yan et al., 2014). Moreover, the levels of miR-192-5p were also upregulated in the serum of mice with hepatic ischemia and reperfusion (I/R), and carbon tetrachloride (CCl_4_)-induced liver injury, as well as in patients with hepatic injury. In contrast, the study by Roy et al. reported that the levels of miR-192-5p were downregulated in the injured liver tissues of mice and human subjects, specifically in the hepatocytes, while there are no variations in the levels of miR-192-5p in other non-parenchymal cells. The knockdown of miR-192-5p repressed H_2_O_2_-induced cell death of Hepa1-6 cells by targeting Zeb2, suggesting lower levels of miR-192-5p in hepatocytes after acute liver injury may exhibit a protective action. In this study, it is noteworthy that the serum levels of miR-192-5p were positively correlated with the AST/ALT ratio in TUNEL-positive cells, and the levels of miR-122, a potent marker of liver injury and hepatic cell death (Roy et al., 2016). Therefore, miR-192-5p may be a promising target in predicting acute liver injuries. Besides, it has also been indicated that miR-192-5p is a potential biomarker of drug-induced hepatobiliary injury and biliary hyperplasia in rats (Church et al., 2016). And a recent study by Zhang and coworkers revealed that the levels of miR-192-5p increase in N,N-dimethyl formamide (DMF)-induced hepatotoxicity in mice, and the inhibition of miR-192-5p in HL-7702 cells can alleviate DMF-induced apoptosis. Further studies have demonstrated that miR-192-5p promotes DMF-induced apoptosis by targeting the anti-apoptotic gene, NIN1/RPN12 binding protein 1 homolog (NOB1) (Zhang et al., 2020). Conclusively, miR-192-5p plays an important role in liver injury induced by different pathologic factors, and maybe a potential biomarker.

#### Diseases of the Digestive Tract

Apart from hepatic disorders, studies on miR-192-5p have also been performed in other diseases of the digestive tract, including esophageal, colorectal, and gastric cancers (Della Vittoria Scarpati et al., 2014; Huang et al., 2017; Xie et al., 2019). Esophageal cancer (EC) ranks seventh in terms of incidence (572,000 new cases) and sixth in mortality overall (509,000 deaths), based on the GLOBOCAN 2018 database, which mainly consists of esophageal squamous cell carcinoma (ESCC) and esophageal adenocarcinoma (EAC) (Bray et al., 2018). Notably, the levels of circulating miR-192-5p increased in both ESCC and EAC, suggesting a promising regulatory role of miR-192-5p in EC (Bansal et al., 2014; Huang et al., 2017). Further studies are essential for investigating its function and mechanism in EC. Physiologically, miR-192-5p is more abundantly expressed in mucosal samples from the small intestine than in those of the large intestine, while the expression of ABCC3, an intestinal transporter, exhibits an opposite tendency in the large and small intestines. By employing TargetScan and MicroCosm Targets version 5, it has been revealed that miR-192-5p can bind to the 3′-UTR of ABCC3 mRNA, thereby inhibiting the expression of the ABCC3 protein (Bruckmueller et al., 2017). Given the independent role of miR-192-5p in the intestine, the dysregulation of miR-192-5p may be related to the genesis of intestinal diseases. Guo and coworkers reported that the levels of miR-192-5p is downregulated in the inflamed terminal ileal mucosa of adults with active Crohn’s disease (CD), and is significantly increased following exclusive enteral nutrition (EEN) therapy (Guo et al., 2015). Additionally, the levels of miR-192-5p are reduced in the duodenum of adults with CD, and are negatively correlated with the expression of C-X-C motif chemokine ligand 2 (*CXCL2*) and nucleotide binding oligomerization domain containing 2 (*NOD2)* genes, which are involved in innate and adaptive immunity in Marsh 3C patients (Magni et al., 2014). Intriguingly, the expression of miR-192-5p showed a similar pattern in pediatric celiac disease, however, there are no variations in the expression of *NOD2* and *CXCL2*. Alternatively, the expression of *MAD2L1*, another target gene associated with cell cycle control, is markedly increased. Therefore, miRNA-regulated gene expression may be disparate in patients with celiac disease, depending on the age of presentation (Buoli Comani et al., 2015). It has been demonstrated that the expression of miR-192-5p is higher in cancer tissues compared to that of para-carcinoma tissues in human CRC (Della Vittoria Scarpati et al., 2014). A recent study by Zhao and coworkers demonstrated that the expression of miR-192-5p is downregulated in CRC, and the knockdown of miR-192-5p can compromise the tumor suppressive effect of the sh-lncRNA, FTX, indicating the anti-tumor effect of miR-192-5p in CRC (Zhao et al., 2020a). Additionally, miR-192-5p represses glycolysis by regulating the expression of sushi repeat-containing protein X-linked 2 (SRPX2) in colon cancer cells (Zhao et al., 2018). Intriguingly, the metabolites produced by intestinal microflora can stimulate the expression of miR-192-5p, which subsequently inhibits the proliferation of colon cancer cells via the RhoA-ROCK-LIMK2 pathway (Huang et al., 2020). A recent study by Tavakolian and coworkers demonstrated that the expression of miR-192-5p is significantly reduced in tissues of human gastric cancer, suggesting that miR-192-5p may play an important role in gastric cancer (Tavakolian et al., 2020). Consistently, vital studies have indicated that miR-192-5p plays a role in the resistance of EC and gastric cancer to chemotherapy, which reverses cisplatin resistance by targeting ERCC3 and ERCC4 in gastric cancer lines (Hummel et al., 2014; Xie et al., 2019).

In general, miR-192-5p is distributed in different regions of the digestive tract and has diverse functions in regulating disease progression. But there is still no valid evidence for confirming the role of miR-192-5p in diseases of the digestive system, and further mechanistic studies are needed to perform.

### Diseases of the Circulatory System

Myocardial fibrosis is a feature of various cardiovascular diseases, which may result in heart failure, arrhythmias, and sudden fatality (Zile and Brutsaert, 2002). The levels of circulating miR-192-5p are upregulated in patients with hypertrophic cardiomyopathy with diffuse myocardial fibrosis and atrial fibrillation (AF), implying that miR-192-5p may be a potential regulator of heart diseases (Natsume et al., 2018) (Fang et al., 2015). The cardiac sodium channel, Nav1.5, is encoded by the *SCN5A* gene, and can lead to lethal ventricular arrhythmias, sudden death, and AF, when mutated. A study by Zhao and coworkers confirmed that the levels of miR-192-5p are abnormally increased in human AF tissues, which is accompanied by a marked downregulation in the expression of *SCN5A*/Nav1.5. Mechanistically, miR-192-5p can bind to the 3′-UTR region of SCN5A, and repress the expression of *SCN5A*/Nav1.5, thereby aggravating arrhythmia (Zhao et al., 2015). In hypoxia/reoxygenation (H/R)-induced apoptosis of H9c2 cardiomyocytes, the overexpression of miR-192-5p promoted apoptosis by targeting FABP3, indicating a potential regulatory role of miR-192-5p in I/R-induced myocardial injury (Zhang et al., 2017). Moreover, the expression of the KCNQ1OT1 lncRNA is downregulated in rats with sepsis-induced myocardial injury, accompanied by an increased expression of miR-192-5p. Functionally, the knockdown of miR-192-5p inhibits LPS-induced apoptosis in H9c2 cells by targeting the XIAP protein. Mechanistically, KCNQ1OT1 protects against LPS-induced myocardial injury by regulating the miR-192-5p/XIAP axis (Sun et al., 2020). Moreover, a recent study also revealed that the expression of miR-192-5p is related to apoptosis in murine myocardial infarction (Ma et al., 2018). In short, miR-192-5p could be a promising therapeutic target for treating arrhythmia and cardiac injury induced by various factors.

### Diseases of the Urinary System

#### Kidney Injury

It has been reported that the levels of miR-192-5p are abundant in the renal cortex of rats, and miR-192-5p is preferentially expressed in the proximal tubules than in the glomeruli, which regulates the activity of the β1 subunit of Na^+^/K^+^-ATPase (ATP1B1) in renal tubules (Tian et al., 2008; Mladinov et al., 2013). Given the important role of miR-192-5p in normal kidneys, the disruption of miR-192-5p by various damaging factors may induce the development of kidney disease. In 2014, a study by Kanki and coworkers demonstrated that urinary miR-192-5p is especially upregulated in rats with cisplatin-induced injury to the proximal tubules, and maybe a potential noninvasive biomarker for assessing nephrotoxicity (Kanki et al., 2014). Correspondingly, the levels of miR-192-5p resumed to nearly normal in cisplatin-induced murine acute kidney injury (AKI) following oral therapy with the nanoparticle urolithin A (UA), a gut metabolite of the dietary tannin, ellagic acid (Zou D et al., 2019). Besides, one important study by Chen and coworkers demonstrated that the levels of miR-192-5p increase ectopically in vancomycin (VAN)-induced AKI and in the VAN-treated human renal tubular epithelial cell line, HK-2. Functionally, miR-192-5p silencing in HK-2 repressed VAN-induced apoptosis and caspase activity and the inhibition of miR-192-5p in mice alleviated VAN-induced AKI. Mechanistically, p53 acted as an upstream regulator that modulated the expression of miR-192-5p in response to VAN-induced nephrotoxicity (Chen et al., 2016). More importantly, Zou and coworkers identified that the urinary levels of miR-192-5p are significantly elevated in rats with I/R-induced kidney injury 72 h post-operation, which was earlier than the time of elevation of kidney injury molecule-1 (KIM-1), indicating that miR-192-5p may promote kidney injury (Zou et al., 2017). Interestingly, the levels of miR-192 are increased in the glomeruli of mice with type 1 (streptozotocin-injected) and type 2 (db/db) diabetes, which is accompanied by the upregulation of TGF-β and col1a2. Mechanistic studies have indicated that miR-192-5p promotes TGF-β-induced collagen deposition in diabetic kidneys disease by targeting the E-box repressor, SMAD-interacting protein 1 (SIP1) (Kato et al., 2007). These studies have highlighted that miR-192-5p may serve as a differential regulator of acute and chronic kidney diseases. Contrary to the aforementioned role of miR-192-5p, the study by Baker and coworkers demonstrated that the expression of miR-192-5p is downregulated in the kidney tissues of patients with hypertension and hypertensive nephrosclerosis, and the knockdown of miR-192-5p in either rat or mice exacerbated the hypertension induced by a high-salt diet. Consistently, another study demonstrated that miR-192-5p protects kidneys against hypertension by targeting ATP1B1, thereby regulating the activity of Na^+^/K^+^-ATPase (Baker et al., 2019).

In conclusion, miR-192-5p may serve as a vital promising diagnostic marker of kidney injury, and further studies are necessary for elucidating the precise mechanisms underlying the activity of miR-192-5p. And more detailed studies are necessary for elucidating the reason underlying the contradictions in the levels of miR-192-5p in various kidney diseases.

#### Other Diseases

It has been reported that bladder cancer (BC) is the 10th most common type of cancer globally, with an estimated 549,000 new cases and 200,000 deaths in 2018, which has a strong male predominance(Bray et al., 2018). In 2018, Ji and coworkers reported for the first time that the levels of miR-192-5p are gradually downregulated in human BC tissues as the disease aggravates, and gain-of-function of it can repress the growth of BC cells by targeting the transcription factor Yin Yang 1 (YY1) (Ji et al., 2018). This study suggests that miR-192-5p may be a potential regulator in progression of BC, but there is still no valid data to confirm its effect on BC. Therefore, further studies are necessary for elucidating the function and mechanism underlying the role of miR-192-5p in BC.

### Diseases of the Reproductive System

It is widely acknowledged that breast cancer is the most common malignancy in women reproductive system. According to the global cancer statistics in 2018, there are approximately 2.1 million newly diagnosed female breast cancer cases, suggesting almost 1 in 4 cancer cases among women, which badly threatens the health of women globally (Bray et al., 2018). Breast cancer is frequently treated by targeting estrogen receptor α (ERα) in various patients, including those treated with tamoxifen (Osborne and Schiff, 2011). Notably, a study by Kim and coworkers demonstrated that the levels of miR-192-5p in patients with recurring breast cancer are higher following treatment with tamoxifen. Functionally, miR-192-5p directly reduces the expression of ERα and promotes tamoxifen resistance in ERα-positive breast cancer cells. Interestingly, Kim *et al.* reported that miR-192-5p is regulated by lymphocyte antigen six complex (LY6K), which aggravates the progression of breast cancer by negatively correlating with the expression of ERα (Kim et al., 2016). Moreover, the levels of miR-192-5p are relatively higher in the serum of patients with breast cancer than those of patients with benign disease and MDA-MB-231 triple-negative breast cancer cells, signifying the potential role of miR-192-5p in breast cancer (Chen et al., 2018; Fang et al., 2018). However, a study by Zhang and coworkers demonstrated that miR-192-5p promotes the sensitivity of breast cancer cells to doxorubicin treatment by inducing apoptosis (Zhang et al., 2019). Therefore, it remains controversial whether miR-192-5p can be an oncogene or tumor suppressor in breast cancer, and further studies are needed to perform in order to identify the role of miR-192-5p.

More importantly, recent studies have indicated that apart from breast cancer, the expression of miR-192-5p is also dysregulated in cervical, ovarian, and prostate cancers (Chen ZJ et al., 2019; Kori and Yalcin Arga, 2018; Liu W et al., 2017). Prostate cancer is one of the malignant cancers among men. It is estimated that there is almost 1.3 million new cases of prostate cancer and 359,000 associated deaths globally in 2018, which ranks as the second most frequent cancer and the fifth leading cause of cancer death in men (Bray et al., 2018). Thus, finding an efficient biomarker is necessary for predicting the occurrence of prostate cancer. Fortunately, a recent study by Chen and coworkers reported that the expression of miR-192-5p is aberrantly increased in prostate cancer tissues, and the enhanced expression of miR-192-5p promotes the proliferation of prostate cancer cells *in vitro* (Chen ZJ et al., 2019). Additionally, bioinformatics analyses have revealed that miR-192-5p is associated with ubiquitin-associated and SH3 domain-containing B (UBASH3B), a poor prognostic marker of prostate cancer (Wang et al., 2019). These two studies reveal the possible regulatory role of miR-192-5p in prostate cancer. However, owing to the lack of studies, there is still a controversy regarding the promotive or protective role of miR-192-5p in diseases of the reproductive system, and further studies are needed to clarify the function and mechanism of miR-192-5p.

### Diseases of the Endocrine System

Physiologically, miR-192-5p is abundant in human pancreatic islets and enriched β-cell populations, indicating the potential involvement of miR-192-5p in diabetes and other pancreatic diseases (van de Bunt et al., 2013). Increasing evidence suggests that the miR-192-5p in biofluids, including the serum and urine, is associated with the presence and incidence of diabetes (Argyropoulos et al., 2015; Jaeger et al., 2018). Besides, miR-192-5p may be a target of traditional Chinese medicine. Jiang Tang Xiao Ke (JTXK) Granules a Chinese medicine, has been reported to exert therapeutic effects by altering the expression of numerous miRNAs in KKAy diabetic mice, including the upregulated expression of miR-192-5p (Mo et al., 2017). Additionally, the plasma levels of miR-192-5p are significantly increased in patients with type 2 diabetes on short-term intensive insulin therapy (IIT), which is accompanied by an improvement of β-cell function (Nunez Lopez et al., 2019). Meaningfully, miR-192-5p was reported to be regulated by the circular RNA, HIPK3, in two diabetes-associated metabolic disorders, namely, hyperglycemia and insulin resistance. It has been demonstrated that the expression of miR-192-5p is reduced in the liver of diabetic mice and oleate-treated HepG2 cells. The inhibition of miR-192-5p promotes hepatic steatosis and insulin resistance by targeting FOXO1 (Cai et al., 2019). A study revealed that methane-rich saline alleviated diabetic retinopathy (DR), a common complication of diabetes mellitus (DM), in a streptozotocin-induced rat model of diabetes, by increasing the retinal levels of miR-192-5p, which is associated with apoptosis and the tyrosine kinase signaling pathway (Wu et al., 2015). In general, miR-192-5p could be a candidate biomarker of the prognosis of diabetes. Some encouraging reports indicate that the expression of miR-192-5p is abnormally increased in the serum of patients with pancreatic cancer and is also present in exosomes (Zhou X et al., 2018; Zou X et al., 2019). Therefore, miR-192-5p could be a promising therapeutic target for the treatment of diseases of the endocrine system, necessitating the detection of the serum levels of miR-192-5p.

### Diseases of the Nervous System

Recent studies reveal that miR-192-5p is related to certain diseases of the nervous system, including Alzheimer’s disease (AD), amyotrophic lateral sclerosis, tuberous sclerosis, peripheral nerve injury, and depression (Trelinska et al., 2016; Rahman et al., 2019; Tang et al., 2019; Liu et al., 2020). A study by Tang *et al.* demonstrated that the overexpression of miR-192-5p rescues cognitive impairment and repairs neural function in a murine model of chronic unpredictable mild stress (CUMS)-induced depression. Mechanistically, miR-192-5p suppresses the TGF-β1 signaling pathway by targeting fibulin (Fbln)-2, a key medium for the neurogenic action of TGF-β1 in neural stem cells (Tang et al., 2019). Inconsistent with the protective role of miR-192-5p in depression, the suppressed expression of miR-192-5p could alleviate neuronal apoptosis and promote the recovery and regeneration of peripheral nerve injury by targeting XIAP in rats with injury to the left sciatic nerve (Liu et al., 2020). Baicalin, a constituent of medical herbs, was reported to inhibit 6-hydroxydopamine-induced neurotoxicity by repressing the expression of miR-192-5p in the PC12 cell model of Parkinson’s disease (PD) (Kang et al., 2019). Although emerging roles of miR-192-5p in diverse diseases of the nervous system have been gradually elucidated, further studies are necessary for identifying the precise mechanisms.

### Diseases of the Motor System

It has been reported that rheumatoid arthritis (RA) is a chronic, systemic inflammatory joint disease that affects the motor system and threatens the health of estimated 1.5 million people in U.S adults (Scott et al., 2010). Noteworthily, a recent study by Zheng and coworkers indicated that exosomal miR-192-5p, secreted by bone marrow-derived mesenchymal stem cells (BMSCs), could delay the inflammatory response by suppressing the expression of ras-related C3 botulinum toxin substrate 2 (RAC2) in collagen-induced arthritis (CIA) (Zheng et al., 2020). Moreover, miR-192-5p was reported to inhibit proliferation and induce the apoptosis of human rheumatoid arthritis fibroblast-like synoviocytes (FLS) by targeting caveolin 1, suggesting a pro-inflammatory function and destructive role of miR-192-5p in RA (Li et al., 2017).

It has been reported that osteosarcoma is the most common malignant tumor in children and adolescents, with the high incidence for 2-3 million per year (Biazzo and De Paolis, 2016). Fortunately, the study by Zhou and coworkers indicated that miR-192-5p represses the proliferation, migration, and invasion of osteosarcoma cells by inhibiting ubiquitin-specific protease 1 (USP1), suggesting the important regulatory role of miR-192-5p in osteosarcoma (Zhou S et al., 2018). However, there is still a long way to identify the function and mechanism of miR-192-5p, and further studies are necessary for elucidating the mechanism underlying the role of miR-192-5p in the progression of osteosarcoma.

## Conclusion

Extensive studies on miR-192-5p have provided valuable knowledge regarding the regulatory functions of miR-192-5p and the mechanisms underlying its role in various cell types and numerous human diseases (Table 1). As a conserved and abundant miRNA, miR-192-5p has been widely studied in mammals, and has diverse roles that are mediated by binding to the 3′-UTR of the target mRNA in different diseases. Recent studies have gradually unveiled the novel upstream regulators of miR-192-5p, including circRNA and lncRNA, which may explain why the levels of miR-192-5p differ among various diseases. Controversially, it has been reported that the expression of miR-192-5p is upregulated or downregulated in different human cancers, by acting as an oncogene or tumor suppressor (Table 2). For instance, the majority of studies have suggested that miR-192-5p acts as a tumor suppressor in NSCLC. It has been confirmed that the expression of miR-192-5p is maintained at an inadequate level in the serum of NSCLC, and functional studies have indicated that miR-192-5p suppresses the proliferation, migration, and invasion of lung cancer cell lines by targeting different critical molecules, including c-Myc and TRIM44. Moreover, important studies have demonstrated that curcumin exerts a protective effect in NSCLC by upregulating miR-192-5p in a p53-dependent manner. However, owing to the flexibility of miR-192-5p in different systems of the human body, scientists may hold controversial views regarding miR-192-5p in certain diseases, including HCC and chemotherapy-resistant breast cancer. At present, there are too many studies on miR-192-5p in human diseases, and the functions of miR-192-5p are different in many above reported diseases, which will bring a problem that, for example, systemic knockout or overexpression of miR-192-5p will cause other side effects, rather than specific targeting of a certain disease. Besides, in the study of miR-192-5p, there is a lack of large-sample clinical data, which is unbeneficial to deepen our understanding of the role of miR-192-5p. But we believe that a comprehensive review of miR-192-5p in different human diseases will encourage the clinical investigation and application of miR-192-5p in diagnosing and predicting human diseases.

**TABLE 2:** the expression and role of miR-192-5p in different human cancers. NA, not available.

Human system	Tumor type	MiR-192-5p	Role	References
Respiratory	NPC	Up	NA	Zou et al. (2020)
	NSCLC	Down	Tumor suppressor	Ye et al. (2015)
	LUAD	Up	NA	Xue et al. (2020)
Digestive	HCC	Controversy	NA	Yan-Chun et al. (2017), Gu et al. (2019), and Zhu et al. (2019)
	Esophageal cancer	Up	Oncogene	Bansal et al. (2014), Huang et al. (2017)
	Colorectal cancer	Controversy	NA	Della Vittoria Scarpati et al. (2014), Zhao et al. (2020a)
	Gastric cancer	Down	Tumor suppressor	Xie et al. (2019), Tavakolian et al. (2020)
Urinary	BC	Down	Tumor suppressor	Ji et al. (2018)
Reproductive	Breast cancer	Controversy	NA	Kim et al. (2016), Chen et al. (2018), Fang et al. (2018), and Zhang et al. (2019)
	Cervical cancer	Up	NA	Kori and Yalcin Arga (2018)
	Ovarian cancer	Up	Oncogene	Liu W et al. (2017)
	Prostate cancer	Up	Oncogene	Chen ZJ et al. (2019)
Endocrine	Pancreatic cancer	Up	NA	Zhou X et al. (2018), Zou X et al., 2019
Motor	Osteosarcoma	Down	Tumor suppressor	Zhou S et al. (2018)

## Glossary

ABCC3: ATP binding cassette transporter subfamily C member 3
AD: Alzheimer’s Disease
AF: atrial fibrillation
AKI: acute kidney injury
ALDH3A2: aldehyde dehydrogenase 3 family member A2
ATG7: autophagy related 7
ATP1B1: the β1 subunit of Na+/K+-ATPase
BC: bladder cancer
BMSCs: bone marrow-derived mesenchymal stem cells
CCl4: carbon tetrachloride
CD: Crohn’s disease
CHB: chronic hepatitis B
CIA: collagen-induced arthritis
circRNA: circular RNA
CRC: colorectal cancer
CSCs: cancer stem cells
CUMS: chronic unpredictable mild stress
CXCL2: C-X-C motif chemokine ligand 2
CXCR5: C-X-C chemokine receptor type 5
DM: diabetes mellitus
DMF: N,N-dimethyl formamide
DR: diabetic retinopathy
EAC: esophageal adenocarcinoma
EBV: Epstein–Barr virus
EC: Esophageal cancer
EEN: exclusive enteral nutrition
EMT: epithelial-to-mesenchymal transition
ER: endoplasmic reticulum
ERα: estrogen receptor α
ESCC: squamous cell carcinoma
FLS: fibroblast-like synoviocytes
FOXO1: forkhead box O1
FXR: farnesoid X receptor
H/R: hypoxia/reoxygenation
HCC: hepatocellular carcinoma
HCs: healthy controls
HFD: high-fat diet
hUC-MSCs: human umbilical cord Wharton’s jelly derived mesenchymal stem cell
I/R: ischaemia and reperfusion
IC: inactive-carriers
IIT: intensive insulin therapy
JTXK: Jiang Tang Xiao Ke
KCNQ1OT1: strand/antisense transcript 1
KIM-1: kidney injury molecule-1
lncRNA: long noncoding RNA
LUAD: adenocarcinoma
LUSC: squamous cell carcinoma
LY6K: lymphocyte antigen 6 complex
miRNAs: MicroRNAs
MMP-16: matrix metalloproteinase-16
NAFLD: nonalcoholic fatty liver disease
NASH: nonalcoholic steatohepatitis
NCs: normal controls
NOB1: NIN1/RPN12 binding protein 1
NOD2: nucleotide binding oligomerization domain containing 2
NPC: Nasopharyngeal carcinoma
NSCLC: non-small cell lung cancer
ORF: open reading frame
PD: parkinson’s disease
PFOA: perfluorooctanoic acid
RA: Rheumatoid arthritis
RAC2: ras-related C3 botulinum toxin substrate 2
SCD-1: stearoyl-CoA desaturase 1
SEMA3A: semaphorin 3A
SGBS: Simpson–Golabi–Behmel syndrome
SIP1: SMAD-interacting protein 1
SRPX2: sushi repeat-containing protein X-linked 2
TG: triglyceride
TRIM44: tripartite motif 44
TRIP13: thyroid hormone receptor interactor 13
UA: urolithin A
UBASH3B: ubiquitin-associated and SH3 domain-containing B
USP1: ubiquitin-specific protease 1
VAN: vancomycin
VAT: visceral adipose tissue
XIAP: X-linked inhibitor of apoptosis protein
YY1: Yin Yang 1

## References

[B1] Argyropoulos C. Wang K. Bernardo J. Ellis D. Orchard T. Galas D. (2015). Urinary MicroRNA profiling predicts the development of microalbuminuria in patients with type 1 diabetes. J. Clin. Med. 4, 1498–1517. 10.3390/jcm4071498 26239688 PMC4519802

[B2] Baker M. A. Wang F. Liu Y. Kriegel A. J. Geurts A. M. Usa K. (2019). MiR-192-5p in the kidney protects against the development of hypertension. Hypertension 73, 399–406. 10.1161/HYPERTENSIONAHA.118.11875 30595117 PMC6339564

[B3] Bansal A. Hong X. Lee I. H. Krishnadath K. K. Mathur S. C. Gunewardena S. (2014). MicroRNA expression can be a promising strategy for the detection of Barrett’s esophagus: a pilot study. Clin. Transl. Gastroenterol. 5, e65. 10.1038/ctg.2014.17 25502391 PMC4274369

[B4] Bartel D. P. (2004). MicroRNAs: genomics, biogenesis, mechanism, and function. Cell 116, 281–297. 10.1016/s0092-8674(04)00045-5 14744438

[B5] Biazzo A. De Paolis M. (2016). Multidisciplinary approach to osteosarcoma. Acta Orthop. Belg. 82, 690–698. 29182106

[B6] Bray F. Ferlay J. Soerjomataram I. Siegel R. L. Torre L. A. Jemal A. (2018). Global cancer statistics 2018: GLOBOCAN estimates of incidence and mortality worldwide for 36 cancers in 185 countries. CA Cancer J. Clin. 68, 394–424. 10.3322/caac.21492 30207593

[B7] Bruckmueller H. Martin P. Kahler M. Haenisch S. Ostrowski M. Drozdzik M. (2017). Clinically relevant multidrug transporters are regulated by microRNAs along the human intestine. Mol. Pharm. 14, 2245–2253. 10.1021/acs.molpharmaceut.7b00076 28510455

[B8] Brunetto M. R. Cavallone D. Oliveri F. Moriconi F. Colombatto P. Coco B. (2014). A serum microRNA signature is associated with the immune control of chronic hepatitis B virus infection. PLoS One 9, e110782. 10.1371/journal.pone.0110782 25350115 PMC4211710

[B9] Buoli Comani G. Panceri R. Dinelli M. Biondi A. Mancuso C. Meneveri R. (2015). miRNA-regulated gene expression differs in celiac disease patients according to the age of presentation. Genes Nutr. 10, 482. 10.1007/s12263-015-0482-2 26233308 PMC4522246

[B10] Bushel P. R. Caiment F. Wu H. O’Lone R. Day F. Calley J. (2018). RATEmiRs: the rat atlas of tissue-specific and enriched miRNAs database. BMC Genomics 19, 825. 10.1186/s12864-018-5220-x 30453895 PMC6245813

[B11] Cai H. Jiang Z. Yang X. Lin J. Cai Q. Li X. (2019). Circular RNA HIPK3 contributes to hyperglycemia and insulin homeostasis by sponging miR-192-5p and upregulating transcription factor forkhead box O1. Endocr. J. 67, 397–408. 10.1507/endocrj.EJ19-0271 31875589

[B12] Caserta S. Kern F. Cohen J. Drage S. Newbury S. F. Llewelyn M. J. (2016). Circulating plasma microRNAs can differentiate human sepsis and systemic inflammatory response syndrome (SIRS). Sci. Rep. 6, 28006. 10.1038/srep28006 27320175 PMC4913253

[B13] Chen J. Chen Z. Huang J. Chen F. Ye W. Ding G. (2018). Bioinformatics identification of dysregulated microRNAs in triple negative breast cancer based on microRNA expression profiling. Oncol. Lett. 15, 3017–3023. 10.3892/ol.2017.7707 29435032 PMC5778821

[B14] Chen J. Wang J. Li H. Wang S. Xiang X. Zhang D. (2016). p53 activates miR-192-5p to mediate vancomycin induced AKI. Sci. Rep. 6, 38868. 10.1038/srep38868 27941921 PMC5150818

[B15] Chen Y. P. Chan A. T. C. Le Q. T. Blanchard P. Sun Y. Ma J. (2019). Nasopharyngeal carcinoma. Lancet 394, 64–80. 10.1016/S0140-6736(19)30956-0 31178151

[B16] Chen Z. J. Yan Y. J. Shen H. Zhou J. J. Yang G. H. Liao Y. X. (2019). miR-192 is overexpressed and promotes cell proliferation in prostate cancer. Med. Princ Pract. 28, 124–132. 10.1159/000496206 30544100 PMC6546031

[B17] Chua M. L. K. Wee J. T. S. Hui E. P. Chan A. T. C. (2016). Nasopharyngeal carcinoma. Lancet 387, 1012–1024. 10.1016/S0140-6736(15)00055-0 26321262

[B18] Church R. J. Otieno M. McDuffie J. E. Singh B. Sonee M. Hall L. (2016). Beyond miR-122: identification of MicroRNA alterations in blood during a time course of hepatobiliary injury and biliary hyperplasia in rats. Toxicol. Sci. 150, 3–14. 10.1093/toxsci/kfv260 26614776 PMC5009612

[B19] Della Vittoria Scarpati G. Calura E. Di Marino M. Romualdi C. Beltrame L. Malapelle U. (2014). Analysis of differential miRNA expression in primary tumor and stroma of colorectal cancer patients. Biomed. Res. Int. 2014, 840921. 10.1155/2014/840921 25143946 PMC4128171

[B20] Fang L. Ellims A. H. Moore X. L. White D. A. Taylor A. J. Chin-Dusting J. (2015). Circulating microRNAs as biomarkers for diffuse myocardial fibrosis in patients with hypertrophic cardiomyopathy. J. Transl. Med. 13, 314. 10.1186/s12967-015-0672-0 26404540 PMC4581079

[B21] Fang R. Zhu Y. Hu L. Khadka V. S. Ai J. Zou H. (2018). Plasma MicroRNA pair panels as novel biomarkers for detection of early stage breast cancer. Front. Physiol. 9, 1879. 10.3389/fphys.2018.01879 30670982 PMC6331533

[B22] Fuschi P. Carrara M. Voellenkle C. Garcia-Manteiga J. M. Righini P. Maimone B. (2017). Central role of the p53 pathway in the noncoding-RNA response to oxidative stress. Aging (Albany NY) 9, 2559–2586. 10.18632/aging.101341 29242407 PMC5764393

[B23] Gibeon D. Menzies-Gow A. (2013). Recent changes in the drug treatment of allergic asthma. Clin. Med. (Lond). 13, 477–481. 10.7861/clinmedicine.13-5-477 24115705 PMC4953799

[B24] Gu Y. Wei X. Sun Y. Gao H. Zheng X. Wong L. L. (2019). miR-192-5p silencing by genetic aberrations is a key event in hepatocellular carcinomas with cancer stem cell features. Cancer Res. 79, 941–953. 10.1158/0008-5472.CAN-18-1675 30530815 PMC6397664

[B25] Guo Z. Wu R. Gong J. Zhu W. Li Y. Wang Z. (2015). Altered microRNA expression in inflamed and non-inflamed terminal ileal mucosa of adult patients with active Crohn’s disease. J. Gastroenterol. Hepatol. 30, 109–116. 10.1111/jgh.12644 24910152

[B26] Huang Y. L. Li X. H. Ma H. Yue H. Y. Hu X. Y. (2020). Metabolites of intestinal microflora upregulate miR-192-5p to suppress proliferation of colon cancer cells via RhoA-ROCK-LIMK2 pathway. Eur. Rev. Med. Pharmacol. Sci. 24, 1794–1806. 10.26355/eurrev_202002_20357 32141548

[B27] Huang Z. Zhang L. Zhu D. Shan X. Zhou X. Qi L. W. (2017). A novel serum microRNA signature to screen esophageal squamous cell carcinoma. Cancer Med. 6, 109–119. 10.1002/cam4.973 28035762 PMC5269712

[B28] Hummel R. Sie C. Watson D. I. Wang T. Ansar A. Michael M. Z. (2014). MicroRNA signatures in chemotherapy resistant esophageal cancer cell lines. World J. Gastroenterol. 20, 14904–14912. 10.3748/wjg.v20.i40.14904 25356050 PMC4209553

[B29] Iguchi T. Niino N. Tamai S. Sakurai K. Mori K. (2017). Comprehensive analysis of circulating microRNA specific to the liver, heart, and skeletal muscle of cynomolgus monkeys. Int. J. Toxicol. 36, 220–228. 10.1177/1091581817704975 28460582

[B30] Ingenito F. Roscigno G. Affinito A. Nuzzo S. Scognamiglio I. Quintavalle C. (2019). The role of Exo-miRNAs in cancer: a focus on therapeutic and diagnostic applications. Int. J. Mol. Sci. 20, 4687. 10.3390/ijms20194687 PMC6801421 31546654

[B31] Jaeger A. Zollinger L. Saely C. H. Muendlein A. Evangelakos I. Nasias D. (2018). Circulating microRNAs -192 and -194 are associated with the presence and incidence of diabetes mellitus. Sci. Rep. 8, 14274. 10.1038/s41598-018-32274-9 30250222 PMC6155281

[B32] Jemal A. Siegel R. Xu J. Ward E. (2010). Cancer statistics, 2010. CA Cancer J. Clin. 60, 277–300. 10.3322/caac.20073 20610543

[B33] Ji D. Jiang L. Li Y. (2018). MiR-192-5p suppresses the growth of bladder cancer cells via targeting Yin Yang 1. Hum. Cell 31, 210–219. 10.1007/s13577-018-0201-6 29536411

[B34] Jin H. Qiao F. Wang Y. Xu Y. Shang Y. (2015). Curcumin inhibits cell proliferation and induces apoptosis of human non-small cell lung cancer cells through the upregulation of miR-192-5p and suppression of PI3K/Akt signaling pathway. Oncol. Rep. 34, 2782–2789. 10.3892/or.2015.4258 26351877

[B35] Jonas S. Izaurralde E. (2015). Towards a molecular understanding of microRNA-mediated gene silencing. Nat. Rev. Genet. 16, 421–433. 10.1038/nrg3965 26077373

[B36] Kang C. Wang L. Kang M. Liu X. Fu Y. Gao J. (2019). Baicalin alleviates 6-hydroxydopamine-induced neurotoxicity in PC12 cells by down-regulation of microRNA-192-5p. Brain Res. 1708, 84–92. 10.1016/j.brainres.2018.12.015 30552896

[B37] Kanki M. Moriguchi A. Sasaki D. Mitori H. Yamada A. Unami A. (2014). Identification of urinary miRNA biomarkers for detecting cisplatin-induced proximal tubular injury in rats. Toxicology 324, 158–168. 10.1016/j.tox.2014.05.004 24863737

[B38] Kato M. Zhang J. Wang M. Lanting L. Yuan H. Rossi J. J. (2007). MicroRNA-192 in diabetic kidney glomeruli and its function in TGF-beta-induced collagen expression via inhibition of E-box repressors. Proc. Natl. Acad. Sci. U.S.A. 104, 3432–3437. 10.1073/pnas.0611192104 17360662 PMC1805579

[B39] Kim Y. S. Park S. J. Lee Y. S. Kong H. K. Park J. H. (2016). miRNAs involved in LY6K and estrogen receptor alpha contribute to tamoxifen-susceptibility in breast cancer. Oncotarget 7, 42261–42273. 10.18632/oncotarget.9950 27304060 PMC5173133

[B40] Kori M. Yalcin Arga K. (2018). Potential biomarkers and therapeutic targets in cervical cancer: insights from the meta-analysis of transcriptomics data within network biomedicine perspective. PLoS One 13, e0200717. 10.1371/journal.pone.0200717 30020984 PMC6051662

[B41] Krattinger R. Bostrom A. Schioth H. B. Thasler W. E. Mwinyi J. Kullak-Ublick G. A. (2016). microRNA-192 suppresses the expression of the farnesoid X receptor. Am. J. Physiol. Gastrointest. Liver Physiol. 310, G1044–G1051. 10.1152/ajpgi.00297.2015 27079614

[B42] Kulcheski F. R. Christoff A. P. Margis R. (2016). Circular RNAs are miRNA sponges and can be used as a new class of biomarker. J. Biotechnol. 238, 42–51. 10.1016/j.jbiotec.2016.09.011 27671698

[B43] Kumar S. Sharawat S. K. Ali A. Gaur V. Malik P. S. Kumar S. (2020). Identification of differentially expressed circulating serum microRNA for the diagnosis and prognosis of Indian non-small cell lung cancer patients. Curr. Probl. Cancer 44, 100540. 10.1016/j.currproblcancer.2020.100540 32007320

[B44] Lee Y. Pham T. X. Bae M. Hu S. O’Neill E. Chun O. K. (2019). Blackcurrant (Ribes nigrum) prevents obesity-induced nonalcoholic steatohepatitis in mice. Obesity (Silver Spring) 27, 112–120. 10.1002/oby.22353 30569636

[B45] Li S. Jin Z. Lu X. (2017). MicroRNA-192 suppresses cell proliferation and induces apoptosis in human rheumatoid arthritis fibroblast-like synoviocytes by downregulating caveolin 1. Mol. Cell Biochem. 432, 123–130. 10.1007/s11010-017-3003-3 28321538

[B46] Liang J. J. Cao D. R. Zhang X. W. Liu L. J. Tan Q. Shi S. (2020). miR-192-5p suppresses uterine receptivity formation through impeding epithelial transformation during embryo implantation. Theriogenology 157, 360–371. 10.1016/j.theriogenology.2020.08.009 32861000

[B47] Lim S. M. Hong M. H. Kim H. R. (2020). Immunotherapy for non-small cell lung cancer: current landscape and future perspectives. Immune Netw. 20, e10. 10.4110/in.2020.20.e10 32158598 PMC7049584

[B48] Liu W. Wang S. Zhou S. Yang F. Jiang W. Zhang Q. (2017). A systems biology approach to identify microRNAs contributing to cisplatin resistance in human ovarian cancer cells. Mol. Biosyst. 13, 2268–2276. 10.1039/c7mb00362e 28861582

[B49] Liu X. Cui X. Guan G. Dong Y. Zhang Z. (2020). microRNA-192-5p is involved in nerve repair in rats with peripheral nerve injury by regulating XIAP. Cell Cycle 19, 326–338. 10.1080/15384101.2019.1710916 31944167 PMC7028159

[B50] Liu X. L. Cao H. X. Wang B. C. Xin F. Z. Zhang R. N. Zhou D. (2017). miR-192-5p regulates lipid synthesis in non-alcoholic fatty liver disease through SCD-1. World J. Gastroenterol. 23, 8140–8151. 10.3748/wjg.v23.i46.8140 29290651 PMC5739921

[B51] Liu X. L. Pan Q. Cao H. X. Xin F. Z. Zhao Z. H. Yang R. X. (2019). Lipotoxic hepatocyte-derived exosomal miR-192-5p activates macrophages via Rictor/Akt/FoxO1 signaling in NAFLD. Hepatology 72, 454–469. 10.1002/hep.31050 PMC10465073 31782176

[B52] Lou L. Tian M. Chang J. Li F. Zhang G. (2020). MiRNA-192-5p attenuates airway remodeling and autophagy in asthma by targeting MMP-16 and ATG7. Biomed. Pharmacother. 122, 109692. 10.1016/j.biopha.2019.109692 31918268

[B53] Lu J. Getz G. Miska E. A. Alvarez-Saavedra E. Lamb J. Peck D. (2005). MicroRNA expression profiles classify human cancers. Nature 435, 834–838. 10.1038/nature03702 15944708

[B54] Ma H. Chen P. Sang C. Huang D. Geng Q. Wang L. (2018). Modulation of apoptosis-related microRNAs following myocardial infarction in fat-1 transgenic mice vs wild-type mice. J. Cell. Mol. Med. 22, 5698–5707. 10.1111/jcmm.13846 30589501 PMC6201345

[B55] Magni S. Buoli Comani G. Elli L. Vanessi S. Ballarini E. Nicolini G. (2014). miRNAs affect the expression of innate and adaptive immunity proteins in celiac disease. Am. J. Gastroenterol. 109, 1662–1674. 10.1038/ajg.2014.203 25070052

[B56] Mladinov D. Liu Y. Mattson D. L. Liang M. (2013). MicroRNAs contribute to the maintenance of cell-type-specific physiological characteristics: miR-192 targets Na+/K+-ATPase beta1. Nucleic Acids Res. 41, 1273–1283. 10.1093/nar/gks1228 23221637 PMC3553948

[B57] Mo F. F. An T. Zhang Z. J. Liu Y. F. Liu H. X. Pan Y. Y. (2017). Jiang Tang Xiao Ke Granule play an anti-diabetic role in diabetic mice pancreatic tissue by regulating the mRNAs and MicroRNAs associated with PI3K-Akt signaling pathway. Front. Pharmacol. 8, 795. 10.3389/fphar.2017.00795 29163176 PMC5671979

[B58] Murphy C. R. (2004). Uterine receptivity and the plasma membrane transformation. Cell. Res. 14, 259–267. 10.1038/sj.cr.7290227 15353123

[B59] Mysore R. Zhou Y. Sadevirta S. Savolainen-Peltonen H. Nidhina Haridas P. A. Soronen J. (2016). MicroRNA-192* impairs adipocyte triglyceride storage. Biochim. Biophys. Acta 1861, 342–351. 10.1016/j.bbalip.2015.12.019 26747651

[B60] Natsume Y. Oaku K. Takahashi K. Nakamura W. Oono A. Hamada S. (2018). Combined analysis of human and experimental murine samples identified novel circulating MicroRNAs as biomarkers for atrial fibrillation. Circ. J. 82, 965–973. 10.1253/circj.CJ-17-1194 29398686

[B61] Nielsen K. O. Jacobsen K. S. Mirza A. H. Winther T. N. Storling J. Glebe D. (2018). Hepatitis B virus upregulates host microRNAs that target apoptosis-regulatory genes in an *in vitro* cell model. Exp. Cell. Res. 371, 92–103. 10.1016/j.yexcr.2018.07.044 30059664

[B62] Nunez Lopez Y. O. Retnakaran R. Zinman B. Pratley R. E. Seyhan A. A. (2019). Predicting and understanding the response to short-term intensive insulin therapy in people with early type 2 diabetes. Mol. Metab. 20, 63–78. 10.1016/j.molmet.2018.11.003 30503831 PMC6358589

[B63] Oda S. Takeuchi M. Akai S. Shirai Y. Tsuneyama K. Yokoi T. (2018). miRNA in rat liver sinusoidal endothelial cells and hepatocytes and application to circulating biomarkers that discern pathogenesis of liver injuries. Am. J. Pathol. 188, 916–928. 10.1016/j.ajpath.2017.12.007 29353062

[B64] Oikawa T. (2016). Cancer stem cells and their cellular origins in primary liver and biliary tract cancers. Hepatology 64, 645–651. 10.1002/hep.28485 26849406

[B65] Osborne C. K. Schiff R. (2011). Mechanisms of endocrine resistance in breast cancer. Annu. Rev. Med. 62, 233–247. 10.1146/annurev-med-070909-182917 20887199 PMC3656649

[B66] Pan Y. Sun Y. Liu Z. Zhang C. (2020). miR1925p upregulation mediates the suppression of curcumin in human NSCLC cell proliferation, migration and invasion by targeting cMyc and inactivating the Wnt/betacatenin signaling pathway. Mol. Med. Rep. 22, 1594–1604. 10.3892/mmr.2020.11213 32626956

[B67] Papi A. Brightling C. Pedersen S. E. Reddel H. K. (2018). Asthma. Lancet 391, 783–800. 10.1016/S0140-6736(17)33311-1 29273246

[B68] Pirola C. J. Fernandez Gianotti T. Castano G. O. Mallardi P. San Martino J. Mora Gonzalez Lopez Ledesma M. (2015). Circulating microRNA signature in non-alcoholic fatty liver disease: from serum non-coding RNAs to liver histology and disease pathogenesis. Gut 64, 800–812. 10.1136/gutjnl-2014-306996 24973316 PMC4277726

[B69] Place R. F. Li L. C. Pookot D. Noonan E. J. Dahiya R. (2008). MicroRNA-373 induces expression of genes with complementary promoter sequences. Proc. Natl. Acad. Sci. U.S.A. 105, 1608–1613. 10.1073/pnas.0707594105 18227514 PMC2234192

[B70] Puppo M. Bucci G. Rossi M. Giovarelli M. Bordo D. Moshiri A. (2016). miRNA-mediated KHSRP silencing rewires distinct post-transcriptional programs during TGF-beta-induced epithelial-to-mesenchymal transition. Cell. Rep. 16, 967–978. 10.1016/j.celrep.2016.06.055 27396342

[B71] Qiu L. Wang T. Ge Q. Xu H. Wu Y. Tang Q. (2019). Circular RNA signature in hepatocellular carcinoma. J. Cancer 10, 3361–3372. 10.7150/jca.31243 31293639 PMC6603403

[B72] Rahman M. R. Islam T. Turanli B. Zaman T. Faruquee H. M. Rahman M. M. (2019). Network-based approach to identify molecular signatures and therapeutic agents in Alzheimer’s disease. Comput. Biol. Chem. 78, 431–439. 10.1016/j.compbiolchem.2018.12.011 30606694

[B73] Raut A. Khanna A. (2016). Enhanced expression of hepatocyte-specific microRNAs in valproic acid mediated hepatic trans-differentiation of human umbilical cord derived mesenchymal stem cells. Exp. Cell. Res. 343, 237–247. 10.1016/j.yexcr.2016.03.015 27001466

[B74] Roy S. Benz F. Alder J. Bantel H. Janssen J. Vucur M. (2016). Down-regulation of miR-192-5p protects from oxidative stress-induced acute liver injury. Clin. Sci. 130, 1197–1207. 10.1042/CS20160216 27129188

[B75] Scott D. L. Wolfe F. Huizinga T. W. (2010). Rheumatoid arthritis. Lancet 376, 1094–1108. 10.1016/S0140-6736(10)60826-4 20870100

[B76] Shi X. Sun M. Liu H. Yao Y. Song Y. (2013). Long non-coding RNAs: a new frontier in the study of human diseases. Cancer Lett. 339, 159–166. 10.1016/j.canlet.2013.06.013 23791884

[B77] Sun F. Yuan W. Wu H. Chen G. Sun Y. Yuan L. (2020). LncRNA KCNQ1OT1 attenuates sepsis-induced myocardial injury via regulating miR-192-5p/XIAP axis. Exp. Biol. Med. 245, 153537022090804. 10.1177/1535370220908041 PMC7153215 32102564

[B78] Tan Y. Lin B. Ye Y. Wen D. Chen L. Zhou X. (2015). Differential expression of serum microRNAs in cirrhosis that evolve into hepatocellular carcinoma related to hepatitis B virus. Oncol. Rep. 33, 2863–2870. 10.3892/or.2015.3924 25962820

[B79] Tang C. Z. Yang J. T. Liu Q. H. Wang Y. R. Wang W. S. (2019). Up-regulated miR-192-5p expression rescues cognitive impairment and restores neural function in mice with depression via the Fbln2-mediated TGF-beta1 signaling pathway. FASEB J. 33, 606–618. 10.1096/fj.201800210RR 30118321

[B80] Tavakolian S. Goudarzi H. Faghihloo E. (2020). Evaluating the expression level of miR-9-5p and miR-192-5p in gastrointestinal cancer: introducing novel screening biomarkers for patients. BMC Res. Notes 13, 226. 10.1186/s13104-020-05071-9 32307002 PMC7168809

[B81] Tian Z. Greene A. S. Pietrusz J. L. Matus I. R. Liang M. (2008). MicroRNA-target pairs in the rat kidney identified by microRNA microarray, proteomic, and bioinformatic analysis. Genome Res. 18, 404–411. 10.1101/gr.6587008 18230805 PMC2259104

[B82] Trelinska J. Fendler W. Dachowska I. Kotulska K. Jozwiak S. Antosik K. (2016). Abnormal serum microRNA profiles in tuberous sclerosis are normalized during treatment with everolimus: possible clinical implications. Orphanet J. Rare Dis. 11, 129. 10.1186/s13023-016-0512-1 27680012 PMC5041396

[B83] Tzur G. Israel A. Levy A. Benjamin H. Meiri E. Shufaro Y. (2009). Comprehensive gene and microRNA expression profiling reveals a role for microRNAs in human liver development. PLoS One 4, e7511. 10.1371/journal.pone.0007511 19841744 PMC2760133

[B84] van de Bunt M. Gaulton K. J. Parts L. Moran I. Johnson P. R. Lindgren C. M. (2013). The miRNA profile of human pancreatic islets and beta-cells and relationship to type 2 diabetes pathogenesis. PLoS One 8, e55272. 10.1371/journal.pone.0055272 23372846 PMC3555946

[B85] van der Ree M. H. Jansen L. Kruize Z. van Nuenen A. C. van Dort K. A. Takkenberg R. B. (2017). Plasma MicroRNA levels are associated with hepatitis B e antigen status and treatment response in chronic hepatitis B patients. J. Infect Dis. 215, 1421–1429. 10.1093/infdis/jix140 28368488

[B86] Wang Z. Wang Y. Peng M. Yi L. (2019). UBASH3B is a novel prognostic biomarker and correlated with immune infiltrates in prostate cancer. Front. Oncol. 9, 1517. 10.3389/fonc.2019.01517 32010618 PMC6974685

[B87] Wen Y. Han J. Chen J. Dong J. Xia Y. Liu J. (2015). Plasma miRNAs as early biomarkers for detecting hepatocellular carcinoma. Int. J. Cancer 137, 1679–1690. 10.1002/ijc.29544 25845839

[B88] Woo I. Christenson L. K. Gunewardena S. Ingles S. A. Thomas S. Ahmady A. (2018). Micro-RNAs involved in cellular proliferation have altered expression profiles in granulosa of young women with diminished ovarian reserve. J. Assist. Reprod. Genet. 35, 1777–1786. 10.1007/s10815-018-1239-9 29987422 PMC6150892

[B89] Wu J. Wang R. Ye Z. Sun X. Chen Z. Xia F. (2015). Protective effects of methane-rich saline on diabetic retinopathy via anti-inflammation in a streptozotocin-induced diabetic rat model. Biochem. Biophys. Res. Commun. 466, 155–161. 10.1016/j.bbrc.2015.08.121 26363454

[B90] Xie X. Huang N. Zhang Y. Wei X. Gao M. Li M. (2019). MiR-192-5p reverses cisplatin resistance by targeting ERCC3 and ERCC4 in SGC7901/DDP cells. J. Cancer 10, 1039–1051. 10.7150/jca.25814 30854110 PMC6400793

[B91] Xue X. Wang C. Xue Z. Wen J. Han J. Ma X. (2020). Exosomal miRNA profiling before and after surgery revealed potential diagnostic and prognostic markers for lung adenocarcinoma. Acta Biochim. Biophys. Sin. 52, 281–293. 10.1093/abbs/gmz164 32073597

[B92] Yamamoto M. Singh A. Ruan J. Gauvreau G. M. O'Byrne P. M. Carlsten C. R. (2012). Decreased miR-192 expression in peripheral blood of asthmatic individuals undergoing an allergen inhalation challenge. BMC Genomics 13, 655. 10.1186/1471-2164-13-655 23170939 PMC3598672

[B93] Yan S. Wang J. Zhang W. Dai J. (2014). Circulating microRNA profiles altered in mice after 28 d exposure to perfluorooctanoic acid. Toxicol. Lett. 224, 24–31. 10.1016/j.toxlet.2013.10.017 24459700

[B94] Yan-Chun L. Hong-Mei Y. Zhi-Hong C. Qing H. Yan-Hong Z. Ji-Fang W. (2017). MicroRNA-192-5p promote the proliferation and metastasis of hepatocellular carcinoma cell by targeting SEMA3A. Appl. Immunohistochem. Mol. Morphol. 25, 251–260. 10.1097/PAI.0000000000000296 26580097

[B95] Ye M. Zhang J. Zhang J. Miao Q. Yao L. Zhang J. (2015). Curcumin promotes apoptosis by activating the p53-miR-192-5p/215-XIAP pathway in non-small cell lung cancer. Cancer Lett. 357, 196–205. 10.1016/j.canlet.2014.11.028 25444916

[B96] Zhang D. Wu Y. Sun G. (2018). miR-192 suppresses T follicular helper cell differentiation by targeting CXCR5 in childhood asthma. Scand. J. Clin. Lab. Invest. 78, 236–242. 10.1080/00365513.2018.1440628 29490514

[B97] Zhang Y. He Y. Lu L. L. Zhou Z. Y. Wan N. B. Li G. P. (2019). miRNA-192-5p impacts the sensitivity of breast cancer cells to doxorubicin via targeting peptidylprolyl isomerase A. Kaohsiung J. Med. Sci. 35, 17–23. 10.1002/kjm2.12004 30844143 PMC11900784

[B98] Zhang Y. Huang R. Zhou W. Zhao Q. Lu Z. (2017). miR-192-5p mediates hypoxia/reoxygenation-induced apoptosis in H9c2 cardiomyocytes via targeting of FABP3. J. Biochem. Mol. Toxicol. 31. 10.1002/jbt.21873 27780314

[B99] Zhang Z. Zhu W. Liu Z. Liu Y. Chang C. Jiang H. (2020). Aberrant expression of miRNA-192-5p contributes to N,N-dimethylformamide-induced hepatic apoptosis. J. Appl. Toxicol. 40, 1683–1693. 10.1002/jat.4028 32648274

[B100] Zhao J. Xu J. Zhang R. (2018). SRPX2 regulates colon cancer cell metabolism by miR-192/215 via PI3K-Akt. Am. J. Transl. Res. 10, 483–490. 29511442 PMC5835813

[B101] Zhao K. Ye Z. Li Y. Li C. Yang X. Chen Q. (2020a). LncRNA FTX contributes to the progression of colorectal cancer through regulating miR-192-5p/EIF5A2 Axis. Onco. Targets Ther. 13, 2677–2688. 10.2147/OTT.S241011 32280242 PMC7127817

[B102] Zhao K. Ye Z. Y. Li Y. C. Li C. Y. Yang X. D. Chen Q. (2020b). LncRNA FTX contributes to the progression of colorectal cancer through regulating miR-192-5p/EIF5A2 Axis. Oncotargets Ther. 13, 2677–2688. 10.2147/Ott.S241011 PMC7127817 32280242

[B103] Zhao Y. Huang Y. Li W. Wang Z. Zhan S. Zhou M. (2015). Post-transcriptional regulation of cardiac sodium channel gene SCN5A expression and function by miR-192-5p. Biochim. Biophys. Acta 1852, 2024–2034. 10.1016/j.bbadis.2015.07.016 26209011 PMC4757900

[B104] Zheng J. Zhu L. Iok In I. Chen Y. Jia N. Zhu W. (2020). Bone marrow-derived mesenchymal stem cells-secreted exosomal microRNA-192-5p delays inflammatory response in rheumatoid arthritis. Int. Immunopharmacol. 78, 105985. 10.1016/j.intimp.2019.105985 31776092

[B105] Zhou S. Xiong M. Dai G. Yu L. Zhang Z. Chen J. (2018). MicroRNA-192-5p suppresses the initiation and progression of osteosarcoma by targeting USP1. Oncol. Lett. 15, 6947–6956. 10.3892/ol.2018.8180 29731868 PMC5920969

[B106] Zhou X. Lu Z. Wang T. Huang Z. Zhu W. Miao Y. (2018). Plasma miRNAs in diagnosis and prognosis of pancreatic cancer: a miRNA expression analysis. Gene 673, 181–193. 10.1016/j.gene.2018.06.037 29913239

[B107] Zhu M. X. Wei C. Y. Zhang P. F. Gao D. M. Chen J. Zhao Y. (2019). Elevated TRIP13 drives the AKT/mTOR pathway to induce the progression of hepatocellular carcinoma via interacting with ACTN4. J. Exp. Clin. Cancer Res. 38, 409. 10.1186/s13046-019-1401-y 31533816 PMC6749659

[B108] Zile M. R. Brutsaert D. L. (2002). New concepts in diastolic dysfunction and diastolic heart failure: Part II: causal mechanisms and treatment. Circulation 105, 1503–1508. 10.1161/hc1202.105290 11914262

[B109] Zou D. Ganugula R. Arora M. Nabity M. B. Sheikh-Hamad D. Kumar M. (2019). Oral delivery of nanoparticle urolithin A normalizes cellular stress and improves survival in mouse model of cisplatin-induced AKI. Am. J. Physiol. Ren. Physiol. 317, F1255–F1264. 10.1152/ajprenal.00346.2019 31532243

[B110] Zou P. Zhu M. Lian C. Wang J. Chen Z. Zhang X. (2019). miR-192-5p suppresses the progression of lung cancer bone metastasis by targeting TRIM44. Sci. Rep. 9, 19619. 10.1038/s41598-019-56018-5 31873114 PMC6928221

[B111] Zou X. Wei J. Huang Z. Zhou X. Lu Z. Zhu W. (2019). Identification of a six-miRNA panel in serum benefiting pancreatic cancer diagnosis. Cancer Med. 8, 2810–2822. 10.1002/cam4.2145 31006985 PMC6558458

[B112] Zou X. Zhu D. Zhang H. Zhang S. Zhou X. He X. (2020). MicroRNA expression profiling analysis in serum for nasopharyngeal carcinoma diagnosis. Gene 727, 144243. 10.1016/j.gene.2019.144243 31743768

[B113] Zou Y. F. Wen D. Zhao Q. Shen P. Y. Shi H. Zhao Q. (2017). Urinary MicroRNA-30c-5p and MicroRNA-192-5p as potential biomarkers of ischemia-reperfusion-induced kidney injury. Exp. Biol. Med. 242, 657–667. 10.1177/1535370216685005 PMC5685255 28056546

